# Plasma Protein Biomarkers Associated with Higher Ovarian Cancer Risk in BRCA1/2 Carriers

**DOI:** 10.3390/cancers13102300

**Published:** 2021-05-11

**Authors:** Hee-Sung Ahn, Jung Yoon Ho, Jiyoung Yu, Jeonghun Yeom, Sanha Lee, Soo Young Hur, Yuyeon Jung, Kyunggon Kim, Youn Jin Choi

**Affiliations:** 1 Asan Medical Center, Asan Institute for Life Sciences, Seoul 05505, Korea; zaulim3@gmail.com (H.-S.A.); yujiyoung202@gmail.com (J.Y.); 2 Department of Obstetrics and Gynecology, Seoul St. Mary’s Hospital, College of Medicine, The Catholic University of Korea, Seoul 06591, Korea; hojy2000@catholic.ac.kr (J.Y.H.); sanha.lee0327@gmail.com (S.L.); hursy@catholic.ac.kr (S.Y.H.); jung-yuyeon@catholic.ac.kr (Y.J.); 3 Cancer Research Institute, College of Medicine, The Catholic University of Korea, Seoul 06591, Korea; 4 Convergence Medicine Research Center, Asan Institute for Life Sciences, Asan Medical Center, Seoul 05505, Korea; nature8309@amc.seoul.kr; 5 Department of Biomedical Sciences, University of Ulsan College of Medicine, Seoul 05505, Korea; 6 Convergence Medicine Research Center, Asan Medical Center, Clinical Proteomics Core Laboratory, Seoul 05505, Korea; 7 Asan Medical Center, Bio-Medical Institute of Technology, Seoul 05505, Korea

**Keywords:** liquid biopsy, ovarian cancer, BRCA1/2, plasma, proteome, biomarker, LC-MS/MS, ELISA

## Abstract

**Simple Summary:**

Most hereditary ovarian cancer is associated with BRCA1/2 variants, and risk-reducing salpingo-oophorectomy during the follow-up monitoring of ovarian cancer development in heathy women with the *BRCA1/2* variant reduces ovarian cancer incidence. The aim of this study was to identify plasma protein biomarkers that can indicate an increased risk of developing ovarian cancer using a proteomic approach based on a population of genetic variants. Two identified biomarkers among differentially expressed proteins, SPARC and THBS1, had lower plasma concentrations in healthy *BRCA1/2* variant carriers than in ovarian cancer patients with the *BRCA1/2* variant; concentration of two proteins increased at the onset of ovarian cancer. These protein markers from non-invasive liquid biopsy sampling could be used to help women with the *BRCA1/2* variant determine whether to undergo an oophorectomy that could potentially affect the quality of life.

**Abstract:**

Ovarian cancer (OC) is the most lethal gynecologic malignancy and in-time diagnosis is limited because of the absence of effective biomarkers. Germline *BRCA1/2* genetic alterations are risk factors for hereditary OC; risk-reducing salpingo-oophorectomy (RRSO) is pursued for disease prevention. However, not all healthy carriers develop the disease. Therefore, identifying predictive markers in the *BRCA1/2* carrier population could help improve the identification of candidates for preventive RRSO. In this study, plasma samples from 20 OC patients (10 patients with *BRCA1/2* wild type (_wt_) and 10 with the *BRCA1/2* variant (_var_)) and 20 normal subjects (10 subjects with *BRCA1/2*_wt_ and 10 with *BRCA1/2*_var_) were analyzed for potential biomarkers of hereditary OC. We applied a bottom-up proteomics approach, using nano-flow LC-MS to analyze depleted plasma proteome quantitatively, and potential plasma protein markers specific to the *BRCA1/2* variant were identified from a comparative statistical analysis of the four groups. We obtained 1505 protein candidates from the 40 subjects, and SPARC and THBS1 were verified by enzyme-linked immunosorbent assay. Plasma SPARC and THBS1 concentrations in healthy *BRCA1/2* carriers were found to be lower than in OC patients with *BRCA1/2*_var_. If plasma SPARC concentrations increase over 337.35 ng/mL or plasma THBS1 concentrations increase over 65.28 μg/mL in a healthy *BRCA1/2* carrier, oophorectomy may be suggested.

## 1. Introduction

Ovarian cancer (OC) is the most lethal gynecologic malignancy, likely because of its late diagnosis. Indeed, most cases are diagnosed at stage III—IV and their five-year survival rate is less than 50% [1]. Since OC patients with early stage (I—II) disease have a five-year survival rate of 93% [1], increasing the early detection rate has always been a top priority in OC research. The currently recommended blood screening test for OC is the detection of tumor marker cancer antigen 125 (CA125) [2]. However, previous studies have concluded that annual screening for this factor has not improved the survival outcomes for OC patients [3,4]. A more effective tumor marker is therefore urgently needed. 

It has been reported that ~18% of OC, particularly high-grade serous adenocarcinoma, is caused by germline genetic variants such as *BRCA1/2*, BRCA1-interacting protein C-terminal helicase 1 (*BRIP1*), and *RAD51* paralog D (*RAD51D*) [5,6,7,8]. *BRCA1/2* germline carriers account for ~15% of OC [5]. Because early detection of an OC is difficult, women carrying *BRCA1/2* variants are advised to undergo a risk-reducing bilateral salpingo-oophorectomy (RRSO) for OC prevention [9]. This procedure may decrease the risk of OC by 80% and is thus recommended, once childbearing is completed, by the American College of Obstetricians and Gynecologists, the Society of Gynecologic Oncology, The National Comprehensive Cancer Network, and other bodies [10,11,12,13]. Notably however, RRSO can cause early menopause with associated osteoporosis and cardiovascular risk [14], and there remains a risk of *BRCA1/2*-related primary peritoneal cancer [11]. 

Similar to hereditary OC, most current evidence-based strategies for managing patients with known inherited risks of breast cancer (e.g. germline *BRCA1/2* variants) rely on prophylactic surgeries such as bilateral mastectomies [15]. Recent studies have suggested that RANK (receptor activator of nuclear factor kappa-B) and RANK ligand (RANKL) are associated with breast cancer risk in *BRCA* gene variant carriers and may be used for precision prevention for treatment [16,17]. However, further OC prevention strategies in women with an inherited risk have not been fully explored.

In recent years, there have been diverse proteomic studies associated with OC that have utilized liquid chromatography-mass spectrometry (LC-MS). Most of these prior investigations have mainly focused on new biomarker identification among differentially abundant proteins in blood-derived specimens [18,19,20,21] or in tumor tissues [22,23]. Previous studies on the integration of genes and proteins have also been conducted in OC [24,25,26]. In our present comparative proteomic analysis, we performed nano-LC-MS analyses of plasma samples taken from both healthy subjects and OC patients with and without *BRCA1/2* variants and identified potential new protein biomarkers of this cancer that were subsequently validated using an enzyme-linked immunosorbent assay (ELISA). The aim was to find novel diagnostic biomarkers of inherited OC development.

## 2. Results

### 2.1. Study Design

Blood samples from 40 study participants were collected and their demographic data are described in Table 1. There were no significant differences in a range of factors other than genetic differences due to the family history between the groups with or without the *BRCA1/2* variants. The subjects comprised equal numbers of healthy participants and OC patients with (HP_var_/OC_var,_ n = 10 each) and without (HP_wt_, OC_wt_ n = 10 each) a *BRCA1/2* variant. Among all 20 healthy subjects (HP_Total_), no significant differences were found between the HP_wt_ and HP_var_ groups in terms of age. In the total OC patient cohort (OC_Total_), none of the key clinical factors differed significantly between the OC_wt_ and OC_var_ groups, including age, cancer stage, *BRCA* variant type (Supplementary Methods), preoperative CA125 concentration, or preoperative platelet concentration (Bonferroni corrected *p*-value > 0.05/7 = 0.0071).

### 2.2. Workflow for the LC-MS/MS-Based Biomarker Candidate Screen and ELISA-Based Validation

**Figure 1:**
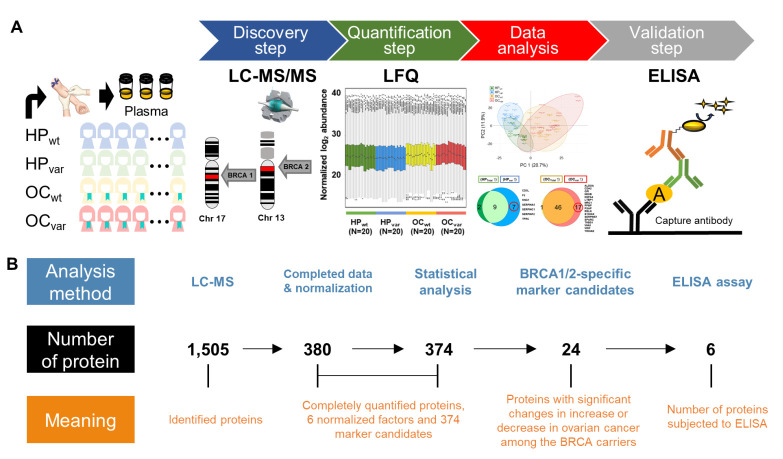
Flow diagram for plasma protein LC-MS/MS-based OC biomarker candidate identification and ELISA-based marker validation. (**A**) Multistage workflow for the identification, quantification, data analysis, and validation steps. Plasma samples from the four study groups (HP_wt_, HP_var_, OC_wt_, and OC_var_) were analyzed by LC-MS/MS after peptide digestion, and the results were quantified and verified by ELISA. (**B**) An initial panel of 1505 proteins was obtained from the initial screening, 380 proteins of which were confirmed by quantification, comprising 374 candidate targets and 6 normalization factors. The Mann–Whitney U test was then used to compare the HP_Total_ vs. OC_Total_ and the HP_var_ vs. OC_var_ groups. In each comparison, we identified proteins with a significant absolute fold-change >2 and Bonferroni-corrected *p*-values < 0.05. We thereby obtained 24 *BRCA1/2*-specific proteins, and six proteins were subjected to ELISA validation.

A workflow was constructed for biomarker identification and validation in the healthy subjects harboring *BRCA1/2* variants who could potentially develop OC (Figure 1A). To screen for *BRCA1/2* variant-specific biomarker candidates, we first identified any differentially abundant plasma proteins between our HP_Total_ and OC_Total_ populations and then subdivided each group into HP_var_, OC_var,_ HP_wt_, and OC_wt_. We obtained 1505 plasma proteins from the initial screening, from which 380 candidate proteins were identified from all samples upon more stringent filtering. We then used six endogenous factors (C2, C6, CFH, CFI, LCP1, and SERPINA7) to normalize the protein abundance levels (Figure S1a) and found that 238 proteins showed a significant positive correlation when comparing these normalized values with the plasma concentrations of the published Plasma Proteome Database [27] (*ρ* = 0.7; Pearson’s correlation coefficient, permutation *p*-value < 0.001; Figure S1B). Statistical analysis was then used to identify *BRCA1/2* variant-specific marker candidates through our strategy. We subsequently validated these candidates by the immunoassay (Figure 1B). 

### 2.3. Proteomic Results from Clinical Plasma Samples by LC-MS/MS

**Figure 2:**
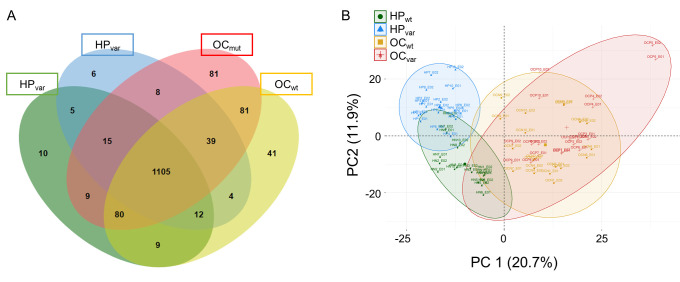
Venn diagram and principal component analysis plot of the identified proteins in the clinical samples. (**A**) Venn diagram of the number of identified proteins in the four study groups (HP_wt_, HP_var_, OC_wt_, and OC_var_; *n* = 10 each). (**B**) Principal component analysis of the plasma proteome using the 40 clinical samples (duplicated runs).

Plasma samples from all 40 study participants were used for the measurement of constitutive proteins via duplicate LC-MS/MS runs. A total of 1505 proteins were identified from 80 LC-MS/MS measurements across the four study groups (HP_wt_, HP_var_, OC_wt_, and OC_var_; Figure 2A and Table S1). From this initial panel of plasma proteins, 380 were completely obtained using a label-free quantification method. These included six relatively stable abundant proteins, C2, C6, CFH, CFI, LCP1, and SERPINA7, that were used to normalize the raw abundance of the other candidates, as detailed in the methods section. Accordingly, 374 normalized proteins were used in the next step analysis (Table S2). By principal component analysis, HP_Total_ and OC_Total_ groups were clearly divided by the first principal component, and the HP_Total_ group could be stratified into HP_wt_ and HP_var_ by PC2, but this was not the case for the OC_Total_ subjects (Figure 2B). This indicated that the *BRCA1/2* genetic background was related to the second principal component in a healthy state but that this association was lost after the development of OC. Next, we present a statistical analysis using different groups of comparison samples in two scenarios (Section 2.4 and Section 2.5) and identify the *BRCA*-specific plasma biomarkers used in this study (Section 2.6).

### 2.4. Scenario I: Differentially Abundant Plasma Proteins between the Total Cohorts of Healthy Subjects and Ovarian Cancer Patients

**Figure 3:**
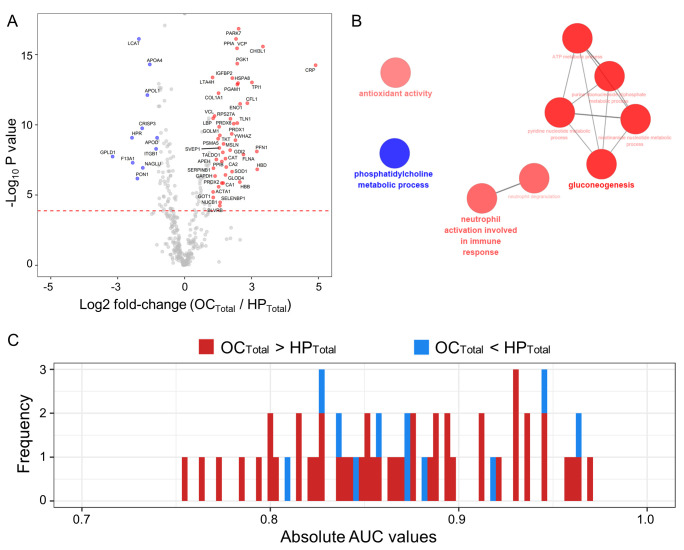
Comparative analysis of the OC_Total_ and HP_Total_ groups using volcano plots, gene ontology (GO) functional annotation, and AUC histograms. (**A**) Volcano plots displaying the mean difference in plasma proteome abundance between the healthy subjects and OC patients (*n* = 20 for each). The indicated *p*-values were calculated using a Mann–Whitney U test. Blue circles denote 11 plasma proteins that showed a significant decrease in the HP samples (Log2 fold-change < ‒1 and Bonferroni-corrected *p*-values < 0.05). Red circles indicate 47 plasma proteins that displayed significant increases in the OC patients (Log2 fold-change > 1 and Bonferroni-corrected *p*-values < 0.05). Gray circles highlight the plasma proteins that did not show statistically significant differences. (**B**) GO analysis of differentially abundant proteins (DAPs). A functional GO network is shown displaying the grouping of biological process terms enriched for HP up-regulated proteins (blue circles) and OC up-regulated proteins (red circles). (**C**) Histogram of AUC values determined from univariate ROC analysis of 58 significant proteins highlighted by volcano analysis.

We compared the abundance of the 374 normalized candidate proteins between the HP_Total_ and OC_Total_ populations using the Mann–Whitney U test, a non-parametric test for comparison. A volcano plot representing the log2-fold-changes (OC_Total_/HP_Total_) against the minus log10-adjusted *p*-values identified 11 proteins as being upregulated in the HP_Total_ and 47 proteins in the OC_Total_ groups (|log2 fold-change| > 1; Bonferroni-corrected *p*-values < 0.05; Figure 3A and Table S3). These differentially abundant proteins (DAPs) in the plasma were linked to known biological processes. Downregulated proteins in the OC_Total_ group were highly involved in phosphatidylcholine metabolic processes, and upregulated proteins in this population were linked to the neutrophil activation involved in immune responses, gluconeogenesis, and antioxidant activity in the ClueGo tools (FDR < 0.01; Figure 3B). We next conducted univariate receiver operating characteristic (ROC) analysis of the proteins showing an association with OC (Figure 3C and Table S3). The results indicated a significant relationship for 58 proteins (*p* < 0.05) with five of these candidates (PARK7, LCAT, PPIA, CHI3L1, and VCP) showing an area under the curve (AUC) value greater than 0.95.

### 2.5. Scenario II: Differentially Abundant Plasma Proteins between the Healthy Subjects and Ovarian Cancer Patients Harboring BRCA1/2 Variants

**Figure 4:**
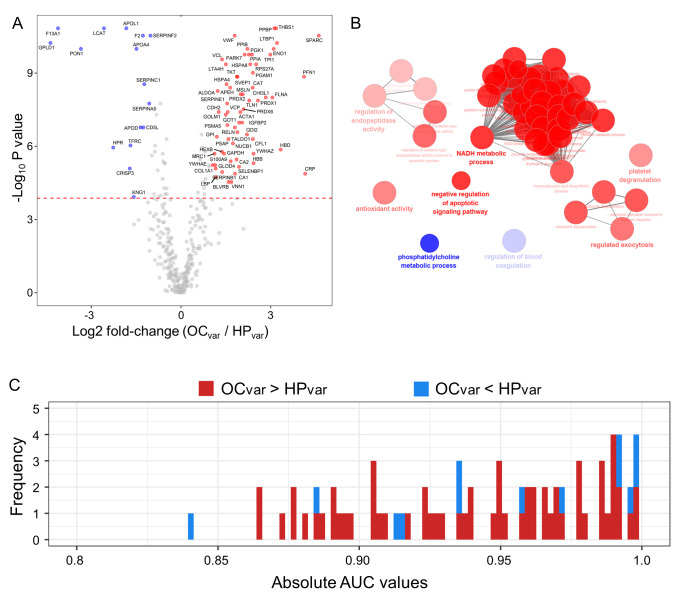
Comparative analysis of the OC_var_ and HP_var_ groups using volcano plots, gene ontology (GO) functional annotation and AUC histograms. (**A**) Volcano plots of the candidate proteins in the HP_var_ and OC_var_ subjects (*n* = 10 each). Blue circles denote 16 proteins showing a significant increase in the HP_var_ subjects (Log2 fold-change < ‒1 and Bonferroni corrected *p*-value < 0.05). Red circles highlight 63 proteins which had significant increases in the OC_var_ patients (Log2 fold-change > 1 and Bonferroni corrected *p*-value < 0.05). Gray circles indicate plasma proteins with no statistically significant differences. (**B**) Functional GO network displaying groupings of biological process terms enriched in the HP_var_ up-regulated proteins (blue circles) and OC_var_ up-regulated proteins (red circles). (**C**) Histogram of AUC values from the univariate ROC analysis of 81 significant proteins identified by volcano analysis.

To identify novel *BRCA1/2* variant-specific markers, we compared the 374 candidate plasma proteins between the HP_var_ and OC_var_ subgroups using a nonparametric test and identified 16 proteins as being upregulated in the HP_var_ group and 63 proteins in the OC_var_ group using a volcano plot (|log2 fold-change (OC_var_/HP_var_)| > 1; Bonferroni corrected *p*-value < 0.05; Figure 4A and Table S4). Using functional gene ontology (GO) analysis, downregulated proteins in the OC_var_ samples were shown to be highly involved in phosphatidylcholine metabolic process and in the regulation of blood coagulation, while upregulated proteins were found to be related to regulation of endopeptidase activity, antioxidant activity, NADH metabolic processes, the negative regulation of apoptotic signaling pathways, platelet degranulation, and regulated exocytosis in the ClueGo tools (FDR < 0.01; Figure 4B). We subsequently conducted univariate ROC analysis against the OC incidence among the *BRCA1/2* carriers (Figure 4C and Table S4) and found that 81 proteins were significant (*p* < 0.05) and had an AUC value of more than 0.8.

### 2.6. Significant Plasma Proteins Associated with the BRCA1/2 Carriers

**Figure 5:**
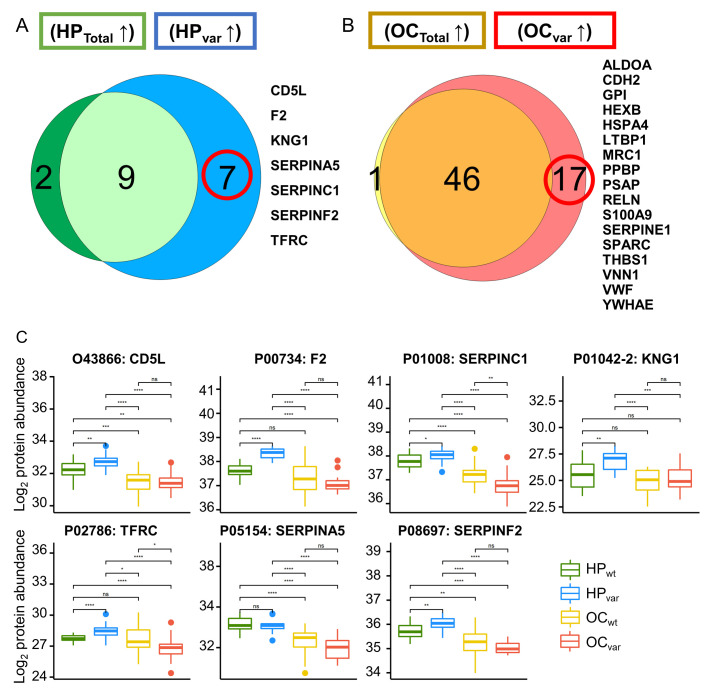

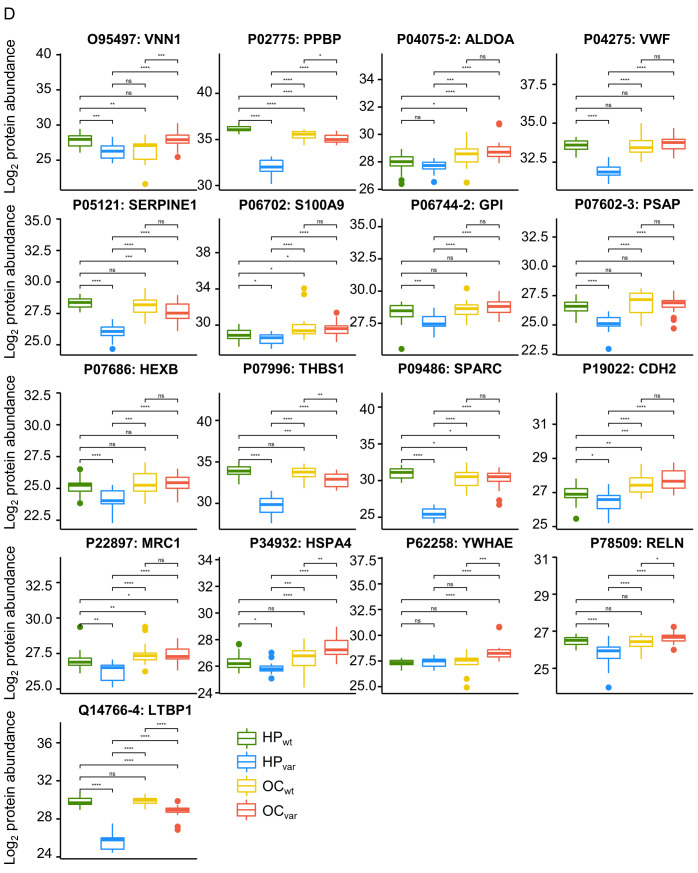
Venn diagrams and boxplots of the significant *BRCA1/2* variant-specific proteins identified in this study. (**A**) Venn analysis of two HP-upregulated DAPs between HP_Total_ vs. OC_Total_ and HP_var_ vs. OC_var_. (**B**) Venn analysis of two OC-upregulated DAPs between OC_Total_ vs. HP_Total_ and OC_var_ vs. HP_var_. (**C**) Boxplots of seven *BRCA1/2* variant-specific HP-upregulated proteins in the four study groups (HP_wt_, HP_var_ OC_wt_, and OC_var_). (**D**) Boxplots of the 17 *BRCA1/2* alteration specific OC-upregulated proteins in the four study groups; * *p* < 0.05, ** *p* < 0.01, *** *p* < 0.001, **** *p* < 0.0001, n.s., not significant.

To identify specific markers of hereditary OC related to a *BRCA1/2* variant, it was necessary to control for confounding factors. The relative complement of DAP’s set of HP_Total_ to DAP’s set HP_var_ and the relative complement of DAP’s set of OC_Total_ to DAP’s set OC_var_ were considered mutually exclusive (Figure 5A,B). The seven HP_var_ up-regulated DAPs are indicated in boxplots for the four groups (Mann–Whitney U Test; Figure 5C). Among these, F2, SERPINC1, and SERPINA5 were found to be related to estrogen procoagulant effects in the Elsevier pathway collection in Enrichr [28]. The association between estrogen and BRCA proteins has been reported previously in breast cancer [29]. Seventeen OC_var_ up-regulated DAPs are presented in boxplots for the four groups (Mann–Whitney U Test; Figure 5D). Among these candidates, SERPINE1, LTBP1, and THBS1 are involved in TGF-beta receptor signaling, which was reported previously to be regulated by the *BRCA* gene [30] and to induce “BRCAness” in breast cancer [31]. Moreover, SPARC, SERPINE1, and THBS1 are reported in the Wikipathways 2019 human database to play a role senescence and autophagy in cancer [32].

### 2.7. Validation by ELISA

**Figure 6:**
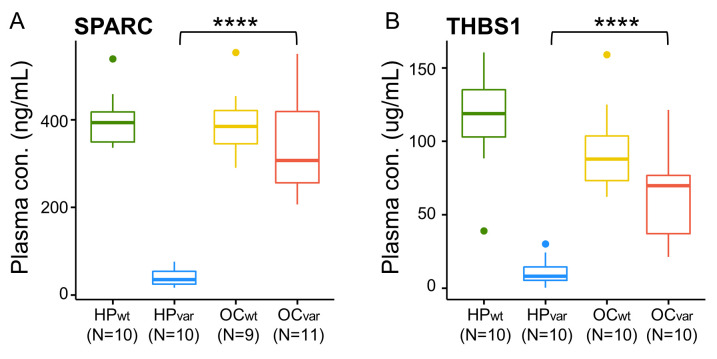
Plasma concentrations of (**A**) SPARC and (**B**) THBS1 in the four groups (HP_wt_, HP_var_, OC_wt_, and OC_var_) determined by ELISA; **** *p* < 0.0001.

From among the 24 specific marker candidates of hereditary OC related to a *BRCA1/2* variant (Figure 5), we selected six proteins for further validation by ELISA based on their functional associations and performance in the validation set (*n* = 80). In the assay results, five of these proteins were significantly different between HP_var_ and OC_var_, the exception being SERPINA5 (Figure S2a. Among these factors, the abundance patterns of two proteins, SPARC and THBS1, in the four study groups showed similarity with MS-based quantification (Figure 5D and Figure 6), whereas the other two proteins, SERPINC1, MRC1, seemed to be different in this regard (Figure S2b–d). Interestingly, the AUC values of SPARC and THBS1 were 1.0 and 0.97 in the ROC analysis between HP_var_ and OC_var_. In the case of SPARC, its plasma concentration could be divided into HP_var_ and OC_var_ sufficiently, and there was an 8.4-fold difference in the quantitative mean (39.87 vs. 337.35 ng/mL). In the case of THBS1, its plasma concentration in the HP_var_ subjects (11.29 μg/mL) was significantly lower than that in the OC_var_ group (65.28 μg/mL), and there was a 5.78-fold difference between the quantitative means. No significant difference in quantification due to the *BRCA1* and *BRCA2* variants within the HP_var_ or OC_var_ groups was observed in the two proteins (*p*-value > 0.05; Figure S3a–d).

## 3. Discussion

Using our biomarker discovery workflow system, we initially identified candidate protein biomarkers of a higher hereditary OC risk in *BRCA1/2* carriers using LC-MS/MS, and later selected six of these proteins for validation by ELISA. These two methods have advantages and disadvantages [33]. The core advantage of LC-MS/MS is its ability to identify and quantify the abundance of hundreds of plasma proteins with high specificity, but this will include a substantial proportion of false-positive results, even if statistically corrected using the Bonferroni correction [34,35,36,37]. ELISA has the advantage of high sensitivity but would be costly if measuring multiple proteins and is limited by the specificity of the antibodies used. In our current results, four proteins, SERPINC1, CDH2, MRC1, and SERPINA5, had inconsistent results between these methods, but two further proteins, SPARC and THBS1, had consistent findings regardless of the measurement technique, indicating a more robust reliability. Moreover, these two validated protein biomarker candidates for OC have been previously associated with tumorigenic mechanisms. Although the role of SPARC has not yet been fully elucidated, most previous studies have suggested it to be a potential oncogene [38,39]. SPARC has also been associated with tumor cell proliferation and migration, the epithelial-mesenchymal transition, and the promotion of the tumor microenvironment [39,40,41,42]. In addition, a further report has suggested that SPARC may have SPARC-null mice accompany the lack of immune response [38,43]. With regard to THBS1, its plasma levels have been previously associated with OC, whereby a higher concentration is linked to improved survival in these patients [44]. In another study involving patient-derived ovarian carcinoma xenografts, the lower expression of THBS1 in tumor cells was identified as an important factor [45].

We observed some biological aberrations potentially relevant to OC among our study subjects harboring *BRCA1/2* variants at the level of the plasma proteome. Through GO analysis, we conducted two statistical tests (HP_Total_ vs. OC_Total_ and HP_var_ vs. OC_var_; Figure 3B and Figure 4B) and identified a *BRCA1/2* variant-specific OC mechanism, i.e., the negative regulation of an apoptotic signaling pathway [46,47,48]. Prior studies of OC patients have found that antioxidant activity is upregulated and that this is related to first-line anticancer drug treatment [49,50]. In addition, human epithelial ovarian carcinoma cells have been shown to have an activated phosphatidylcholine mechanism [51]. General cancer mechanisms have also been described such as Warburg-effect-related gluconeogenesis [52,53] and neutrophil activation involved in immune responses [54,55,56,57]. 

In addition, 8–17% of OC are associated with *BRCA1/2* variations, whereas 51-54% are associated with breast cancer [58]. Osteoprotegerin, a RANKL inhibitor, has been studied as a predictive biomarker for hereditary breast cancer [16,59]. Among *BRCA1/2* carriers, however, reliable methods to predict the risk of OC have been lacking [58]. The locations of variants in *BRCA1/2* and their contribution to the risk of developing OC have been identified in many studies [58,60,61]. Risk management strategies in *BRCA1/2* variant-positive women mainly involve chemoprevention, RRSO, and periodic surveillance [62,63,64]. Inexpensive oral contraceptives were also recommended and are known to reduce the risk of OC by 40–50% [65,66]. However, they increased the thromboembolic risk as well as the risk of breast cancer development among *BRCA1/2* carriers [67,68]. RRSO is another viable approach to OC prevention in *BRCA1/2* variant-positive women. This intervention almost completely decreased the risk of cancer but would cause menopause, and the risk of primary peritoneal cancer still remained. NCCN guidelines thus recommend that *BRCA1/2* carriers undergo this procedure after childbearing under consultation with an expert (https://www.nccn.org, accessed on 11 May 2021). It must be noted however that RRSO has limitations, that surgical decisions are generally complicated, and that the patients will need subsequent hormone therapy, which has quality of life implications. Post-RRSO cases can also suffer from menopausal symptoms, which may further decrease their quality of life. The recommended age range for RRSO is late 30s to early 40s for *BRCA1/2* carriers. Notably however, the number of women undergoing this procedure during the recommended periods has been decreasing because of the higher proportion of women electing to have children in their 30s and 40s [69,70]. 

A final preventative strategy for OC onset in *BRCA1/2* variant-positive women involves a periodic examination protocol consisting of an ultrasound examination and measurement of CA-125 in the blood. This strategy has a high false-positive rate however, and it is fundamentally difficult to block the development of cancer in this way. Two diagnostic methods affect mortality in accordance with a woman’s age. Although it greatly reduced cancer incidence, it was not effective in menopausal women. Our new markers, SPARC and THBS1, could possibly replace CA-125 in periodic testing strategies in these cases. Unlike CA-125, which increases the quantitative value in the blood when cancer occurs, SPARC shows low variation between different genetic backgrounds and is highly expressed upon OC onset. Genetic effects and cancer occurrence can thus be considered at the same time, and the rate of false-positives can be reduced using this biomarker. Hence, screening for plasma SPARC and THBS1 may be a more reliable method of selecting candidates for RRSO and for predicting germline OC occurrence. 

**Figure 7:**
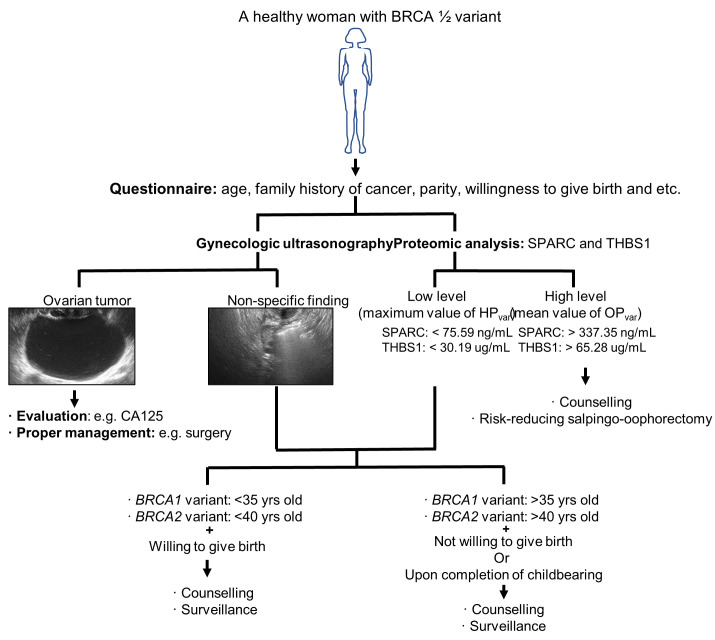
Suggested clinical management approach for healthy women harboring *BRCA1/2* variants that confer a high risk of OC. Step-by-step tree-based guidelines are indicated.

We have formulated a decision-tree for healthy women with *BRCA1/2* variants, based on our current findings (Figure 7). These women are recommended to first respond to a questionnaire and provide information such as age, family history of cancer, etc., and then undergo gynecologic ultrasonography and proteomic analysis of SPARC and THBS1. If the gynecologic ultrasonogram reveals the presence of ovarian tumors, a full evaluation and clinical management regimen must be implemented using current best-practice guidelines [71]. If the results of the gynecologic ultrasonography show non-specific findings or low levels of SPARC/ THBS1 (maximum value of HP_var_, cutoff (ng/mL or μg/mL) = 75.59/30.19), we recommend counseling for the patients including consideration of an RRSO intervention. If the plasma protein levels of SPARC/ THBS1 are high (mean value of OC_var_, cut off= 337.35/65.28), we recommend counseling and careful surveillance.

Our study with a plasma proteomic analysis focus had some limitations of note. First, the patient population was homogeneous and small. Each study group (HP_wt_, HP_var_, OC_wt_, and OC_var_) included only 10 participants. However, there were significant differences between the groups in terms of the DAPs that we selected in scenarios I and II, and the sample size was thus sufficient (Tables S3 and S4). Future multicenter studies or the involvement of international consortia such as the Consortium of Investigators of Modifiers of *BRCA1/2* (CIMBA) (http://cimba.ccge.medschl.cam.ac.uk/, accessed on 11 May 2021) is warranted to validate our biomarkers and to identify others. In addition, the age range of our healthy participants was 32–42 and that of our OC patients was 57–59 (Table 1). However, our proteomic analyses indicated that the plasma levels of SPARC and THBS1 did not correlate with age (*ρ* = 0.086; SPARC, *ρ* = 0.095; THBS1, Spearman correlation coefficient). In addition, the mechanisms underlying OC development include not only germline variants but also somatic variants, a loss of heterozygosity or an allelic deletion, and epigenetic modifications [72]. Other factors can thus contribute to the development of OC independently of germline variants. This should also be a focus of future efforts to identify predictive biomarkers of this cancer. In this study, no differences of abundance of protein markers were noticed between the OC patients with *BRCA1* and *BRCA2* variants. Our data were supported by previous studies and clinical practices performed in real-world *BRCA1/2* target therapies (poly(adenosine diphosphate–ribose) polymerase inhibitors; olaparib and niraparib) and conventional chemotherapies, which are used regardless of *BRCA1* or *BRCA2* status [73,74,75,76,77].

## 4. Materials and Methods 

### 4.1. Sample Subjects

All specimens used in this study were obtained with appropriate consent and with the approval of the Institutional Review Board of Seoul St. Mary’s Hospital, the Catholic University of Korea, College of Medicine (IRB number: KC17TESI0690). Plasma samples were obtained preoperatively from 20 OC patients and 20 HPs. A total of 84 plasma samples were obtained for ELISA verification (Table S5). Plasma samples from OC patients were obtained from Seoul St. Mary’s Hospital and the Korean Gynecologic Cancer Bank and from healthy subjects during a medical checkup at Seoul St. Mary’s Hospital. All samples were snap-frozen in liquid nitrogen and stored at −80 °C until analysis.

### 4.2. Sample Preparation

Plasma samples were prepared sequentially through steps of high abundant plasma protein depletion and trypsin/Lys-C digestion. Initially, fourteen high concentration plasma proteins were depleted through a Human 14 Multiple Affinity Removal (100 × 4.6 mm; MARS14, Agilent, CA, USA) column mounted on an HPLC system (Shimadzu LC20AT HPLC system, Shimadzu LTD, JP). Depleted proteins were digested into peptides using the amicon-adapted enhanced FASP method [78] and salt was removed by the C18 desalting cartridge (Sep-Pak C18 1 cc, Waters, MA, USA). For details, refer to previously published papers [79,80]. 

### 4.3. Nano-LC-ESI-MS/MS Analysis

Peptide separation was performed using the Dionex UltiMate 3000 RSLCnano system (Thermo Fisher Scientific, Waltham, MA, USA). The dried samples were reconstituted with 25 μL of 0.1% formic acid, and 5 μL were injected into a C18 Pepmap trap column (20 mm × 100 μm i.d., 5 μm, 100 Å; Thermo Fisher Scientific) and separated by an Acclaim™ Pepmap 100 C18 column (500 mm × 75 μm i.d., 3 μm, 100 Å; Thermo Fisher Scientific) over 200 min (350 nl/min) using a 0–48% acetonitrile gradient in 0.1% formic acid and 5% DMSO for 150 min at 50°C. The LC was coupled to a Q Exactive mass spectrometer (Thermo Fisher Scientific) with a nano-ESI source. Mass spectra were acquired in a data-dependent mode with an automatic switch between a full scan with 20 data-dependent MS/MS scans. The target value for the full scan MS spectra was 3,000,000 with a maximum injection time of 100 ms and a resolution of 70,000 at m/z 400. The ion target value for MS/MS was set to 1,000,000 with a maximum injection time of 50 ms and a resolution of 17,500 at m/z 400. Dynamic exclusion of repeated peptides was applied for 20 s. All MS data have been deposited in the Proteomics Identificiations Database (PRIDE) archive [81] under PXD023508. 

### 4.4. Database Searching and Label-Free Quantitation 

The acquired MS/MS spectra were retrieved using the SequestHT on Proteome discoverer (version 2.2, Thermo Fisher Scientific) against the SwissProt human protein sequence database (May 2017). Briefly, precursor mass tolerance was set to ± 10 ppm and MS/MS tolerance was set at 0.02 Da. The search parameters were set as default including cysteine carbamidomethylation as a fixed modification and N-terminal acetylation and methionine oxidation as variable modifications with 2 miscleavages. The false discovery rates were set at 1% for the peptides in each analysis using “Percolator” [82]. From the SEQUEST search output, peptide filters that included peptide confidence, peptide rank, score versus charge state, and search engine rank were set at the default values for the proteome discoverer. Label-free quantitation was performed using the peak intensity for the unique and razor peptides of each protein and excluded peptides including methionine oxidation. 

### 4.5. Normalization of Raw LC-ESI-MS/MS Data

The raw protein abundances of the selected six normalizing proteins, C2, C6, CFH, CFI, LCP1, and SERPINA7, in each sample were divided by the corresponding median value of all samples. The geometric mean of the six ratios of the sample was then used as the normalization scaling factor (NSF) for that sample. The details of this method are also provided in previous studies [83,84].

### 4.6. ELISA

For each target protein detection, plasma samples were diluted to the same level as the sample diluent provided in the ELISA kit. The following kits were used for each protein: SERPINA5 (MBS938556, MyBioSoure, San Diego, CA, USA), SERPINC1 (DSPC10, R&D Systems, Minneapolis, MN, USA), CDH2 (DY1388-05, R&D Systems), MRC1 (MBS2019261, MyBioSoure), SPARC (DSP00, R&D Systems), THBS1 (MBS701627, MyBioSoure). The ELISA procedures were performed in each case in accordance with the manufacturer’s instructions with no modifications.

### 4.7. Statistical Analyses

We used two types of Venn diagram drawing tool, jvenn [85] and Venn Diagram Plotter [86] Data were also analyzed using RStudio (version 1.1.456, Boston, MA, USA) including R (version 3.6.0, Vienna, Austria). The statistical R software packages used included ggplot2 for drawing histograms and volcano plots, ggpubr for drawing boxplots, stats for applying the Mann–Whitney test, pcamethods for the PCA analysis, and WMWssp for minimal sample size calculation for the Mann–Whitney U test. 

### 4.8. Pathway Analysis

ClueGo (version 2.5.1) [87] was used to analyze differentially abundant proteins between OC_Total_ and HP_Total_ and between OC_var_ and HP_var_ study participants. This software was plugged into the Cytoscape (version 3.6.1) [88]. Searches were conducted for GO biological processes only. To group GO terms, the kappa score was set at 0.4 and the number of overlapping genes to combine groups was set at 50%.

## 5. Conclusions

Effective follow-up monitoring for OC occurrence in *BRCA1/2* variant carriers at high risk can be conducted through plasma biomarker-based diagnostic tests.

## Figures and Tables

**Table 1:** Demographics and disease characteristics of the study subjects used for nano-LC-ESI-MS/MS analysis.

Characteristics	Healthy Subjects (HP_Total_)			Ovarian Cancer Patients (OC_Total_)		
	HP_wt_ (N = 10)	HP_var_ (N = 10)	*p*-Value	OC_wt_ (N = 10)	OC_var_ (N = 10)	*p*-Value
Age (mean ± SD)	42 ± 10.4	32 ± 14.5	0.116	59 ± 6.7	57 ± 9.1	0.622
Concurrent cancer			1.0			0.032
Yes (n)	0	0		0	5	
No (n)	10	10		10	5	
Family history			<0.001			1.0
Yes (n)	0	10		0	1	
No (n)	10	0		10	9	
OC stage						1.0
II	NA	NA		1	0	
III	NA	NA		8	9	
IV	NA	NA		1	1	
*BRCA* variant			1.0			0.650
*BRCA1*	NA	5		NA	3	
*BRCA2*	NA	5		NA	7	
Ovarian histopathology						1.0
High-grade serous carcinoma	NA	NA		10	10	
Preoperative CA125 level (mean ± SD)	NA	NA		3167 ± 5910.9	1662 ± 1793.7	0.451
Preoperative platelet level (mean + SD)	NA	NA		323 ± 157.9	308 ± 215.3	0.855

NA: not applicable, SD: standard deviation. All results are reported as a mean (SD) or percentage (%), with *p*-values appropriately calculated using the Mann–Whitney U test or Fisher’s exact test.

## Data Availability

The data that support the findings of this study are available from the corresponding author upon reasonable request.

## Supplementary Materials

The following data are available online at https://www.mdpi.com/article/10.3390/cancers13102300/s1. Figure S1. (**a**) Boxplots of normalized plasma protein abundances determined from duplicate LC-MS/MS runs of the 40 clinical samples (10 ovarian cancer (OC) patients and 10 healthy participants (HP) with or without *BRCA1/2* variants). (**b**) Scatter plot of the normalized log2 abundance and log2 immunoassay concentrations of 238 identified plasma proteins (Pearson correlation coefficient (*ρ*): 0.7 and *p*-value: 9.9 × 10^−5^). Figure S2. Boxplots of plasma protein concentrations determined by ELISA analysis (ng/mL) of SERPINA5 (**a**), SERPINC1 (**b**), CDH2 (**c**), and MRC1 (**d**) in the four study groups (HP_wt_, HP_var_, OC_wt_, and OC_var_); * *p* < 0.05, **** *p* < 0.0001. Figure S3. Boxplots of plasma protein concentrations determined by ELISA analysis of SPARC (ng/mL) in HP_var_ (**a**) and OC_var_ (**b**) and THBS1 (μg/mL) in HP_var_ (**c**) and OC_var_ (**d**) according to *BRCA1* and *BRCA2* variants; ns: not significant. Table S1. Number of identified proteins and their raw abundance levels determined from duplicate LC-MS/MS runs with the 40 clinical samples. Table S2. Normalized abundance levels of 374 proteins determined from 80 LC-MS/MS runs. Table S3. Differentially abundant proteins between the healthy subjects and ovarian cancer patients. Table S4. Differentially abundant proteins between healthy subjects and ovarian cancer patients all carrying *BRCA1/2* variants. Table S5. A total of 80 plasma samples obtained for six ELISA verification tests. Supplementary Methods.

## Abbreviations

OC	ovarian cancer
CA125	cancer antigen 125
RRSO	risk-reducing bilateral salpingo-oophorectomy
RANK	receptor activator of nuclear factor kappa-B
RANKL	receptor activator of nuclear factor kappa-B ligand
LC-MS	liquid chromatography-mass spectrometry
ELISA	enzyme-linked immunosorbent assay
HP	healthy participant
SD	standard deviation
LC-MS/MS	liquid chromatography-tandem mass spectrometry
DAP	differential abundant protein
FDR	false discovery rate
ROC	receiver operating characteristic
AUC	area under curve
GO	gene ontology
ESI-LC-MS/MS	electrospray ionization liquid chromatography–tandem mass spectrometry
MARS14	multiple Affinity Removal Column Human 14
ABC	ammonium bicarbonate
