# Supplementary material for: Plasma Protein Biomarkers Associated with Higher Ovarian Cancer Risk in BRCA1/2 Carriers
Source: Cancers (Basel). 2021 May 11;13(10):2300. doi: 10.3390/cancers13102300 (PMC8150736; doi:10.3390/cancers13102300)
Supplement: Supplementary file 1 [file cancers-13-02300-s001.zip › Supplementary Methods.pdf]

Supplementary Methods

Plasma protein biomarkers associated with higher ovarian cancer risk in BRCA1/2 carriers

Hee-Sung Ahn [1], Jung Yoon Ho [2,3], Jiyoung Yu [1], Jeonghun Yeom [4], Sanha Lee [2], Soo Young Hur [2,3], Yuyeon Jung [2,3], Kyunggon Kim [1,5,6,7,*], Youn Jin Choi [2,3,*]

BRCA1/2 variant analysis

We performed targeted NGS using the BRCAaccuTest PLUS panel (NGeneBio Co. Ltd., Seoul, Republic of Korea) and MiSeq instrument (Illumina Inc., San Diego, CA, USA) or Axen BRCA panel (Macrogen, Seoul, Republic of Korea) and Nextseq500 instrument (Illumina Inc.), in accordance with the manufacturers' protocols. After FASTQ files were generated, the Bam file obtained from sequencing was processed with NGeneAnalySys software (NGeneBio Co. Ltd.). Variant calling was performed on the variant call format (VCF) output files by evaluating the coverage (the number of times that targeted, during the sequencing) and the quality score. Then, we filtered only non-synonymous exonic sin-gle-nucleotide variants (SNVs) according to the quality criteria: (1) coverage of at least 20×; (2) a Q-score ≥ 30 (an error rate in base calling of 1 in 1000); and (3) at least 30% of the reads indicating the variant (variant frequency). Copy number variations were also de-tected by calculating the ratio of each of the total amplicons in each sample based on the normalized amplicon coverage derived from other samples within the same run [1,2]. This analysis included read alignment to the human reference genome (Genome Refer-ence Consortium, GRCh37).

Reference

1. Han, E.; Yoo, J.; Chae, H.; Lee, S.; Kim, D.H.; Kim, K.J.; Kim, Y.; Kim, M. Detection of BRCA1/2 large genomic rearrangement including BRCA1 promoter-region deletions using next-generation sequencing. *Clin Chim Acta* **2020**, *505*, 49-54, doi:10.1016/j.cca.2020.02.023.
2. Chan, K.Y.; Ozcelik, H.; Cheung, A.N.; Ngan, H.Y.; Khoo, U.S. Epigenetic factors controlling the BRCA1 and BRCA2 genes in sporadic ovarian cancer. *Cancer Res* **2002**, *62*, 4151-4156.
