# Supplementary material for: Plasma Protein Biomarkers Associated with Higher Ovarian Cancer Risk in BRCA1/2 Carriers
Source: Cancers (Basel). 2021 May 11;13(10):2300. doi: 10.3390/cancers13102300 (PMC8150736; doi:10.3390/cancers13102300)
Supplement: Supplementary file 1 [file cancers-13-02300-s001.zip › Supplementary_figures.pdf]

cancers

MDPI

Supplementary Materials

Plasma Protein Biomarkers Associated with Higher Ovarian Cancer Risk in BRCA1/2 Carriers

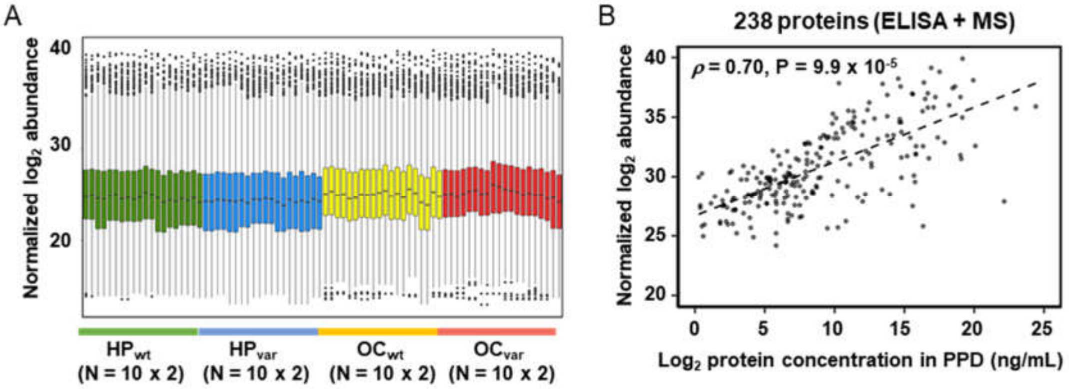

Figure S1. (**A**) Boxplots of normalized plasma protein abundances determined from duplicate LC-MS/MS runs of the 40 clinical samples (10 ovarian cancer (OC) patients and 10 healthy participates (HP) with or without *BRCA1/2* variants). (**B**) Scatter plot of the normalized $\log_2$ abundance and $\log_2$ immunoassay concentrations of 238 identified plasma proteins (Pearson correlation coefficient (ρ): 0.7 and p-value: 9.9×10^{-5}).

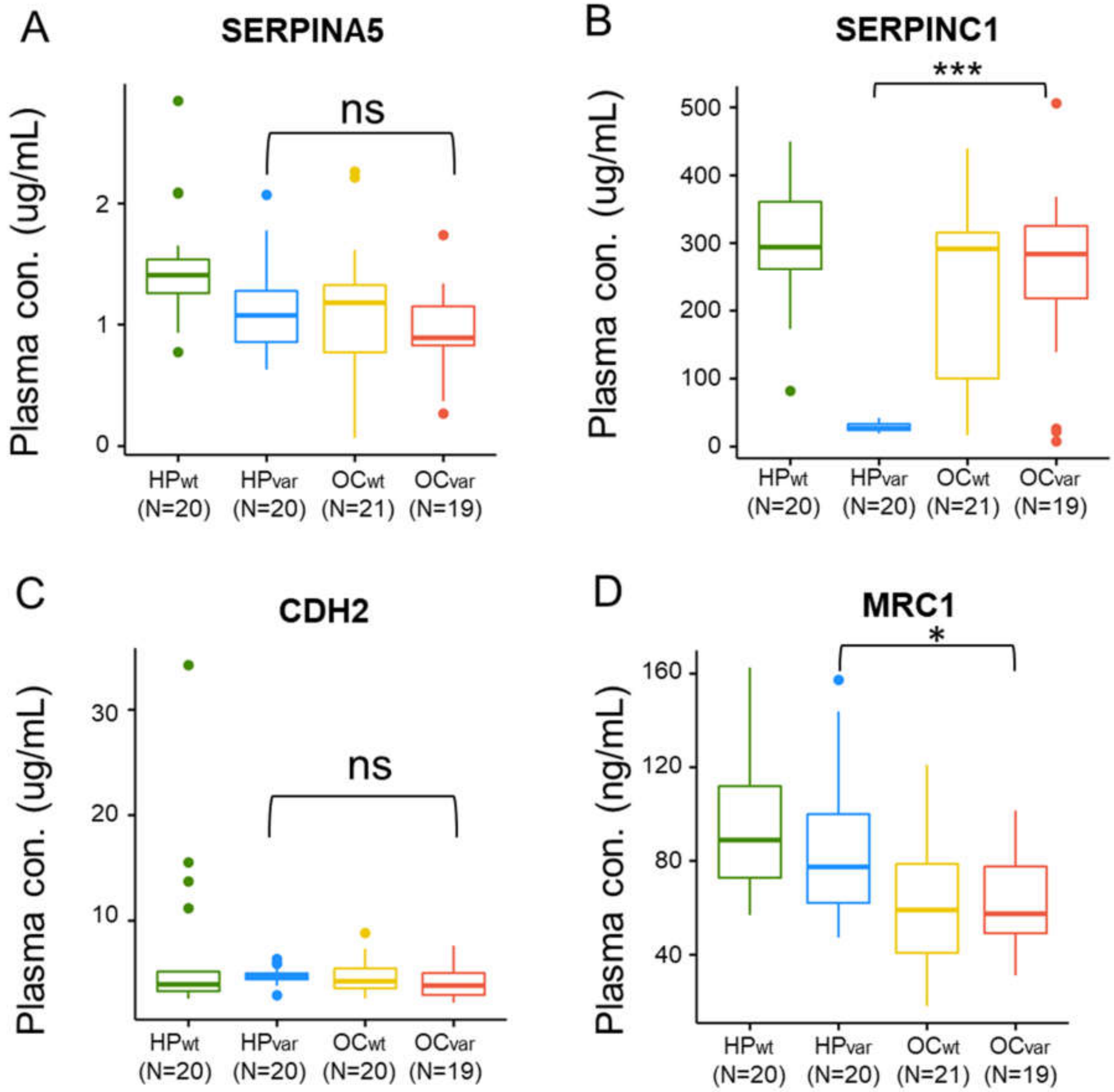

Figure S2. Boxplots of plasma protein concentrations determined by ELISA analysis (ng/mL) of SERPINA5 (**A**), SERPINC1 (**B**), CDH2 (**C**) and MRC1 (**D**) in the four study groups (HP_{wt}, HP_{var}, OC_{wt} and OC_{var}); ns: not significant, * $p < 0.05$, *** $p < 0.001$.

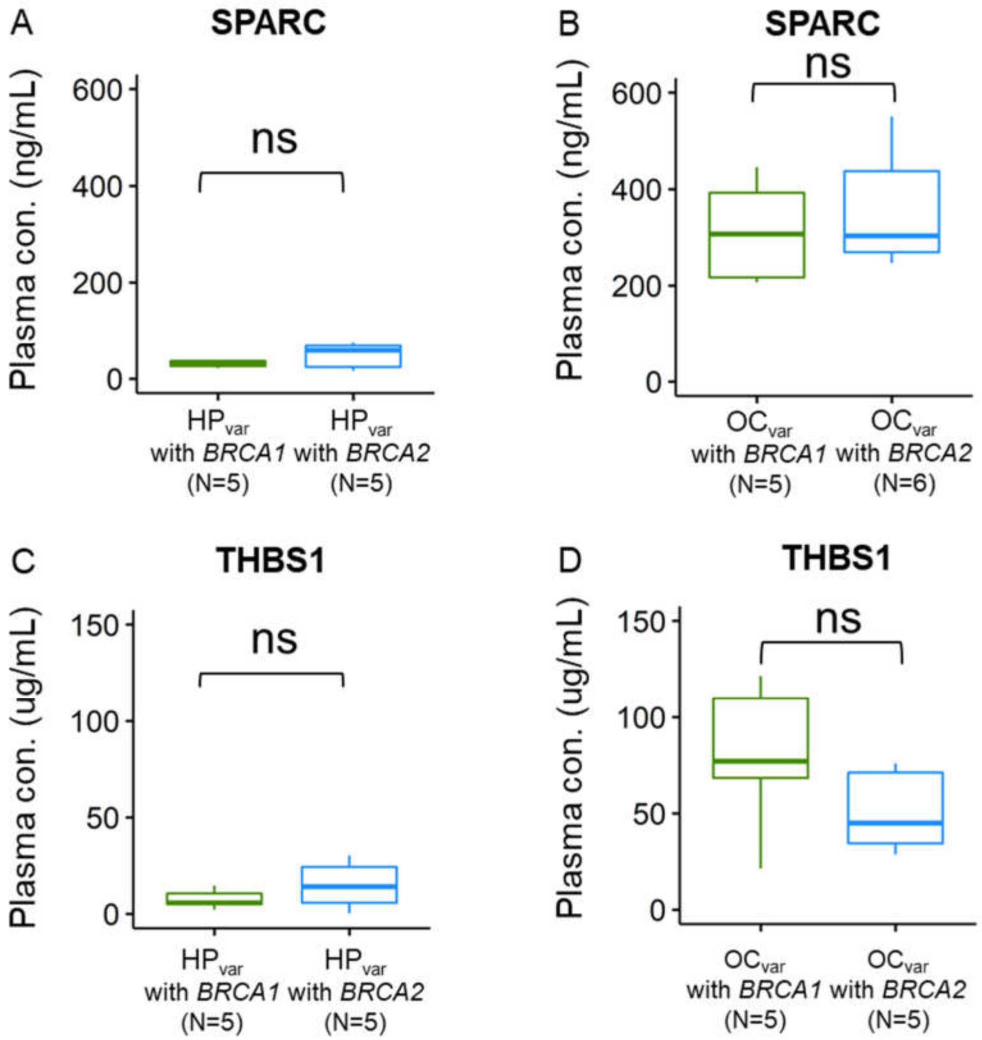

Figure S3. Boxplots of plasma protein concentrations determined by ELISA analysis of SPARC (ng/mL) in HP_{var} (**A**) and OC_{var} (**B**) and THBS1 (ug/mL) in HP_{var} (**C**) and OC_{var} (**D**) according to *BRCA1* and *BRCA2* variants; ns: not significant.
